# Roles of Different Transport Modes in the Spatial Spread of the 2009 Influenza A(H1N1) Pandemic in Mainland China

**DOI:** 10.3390/ijerph16020222

**Published:** 2019-01-14

**Authors:** Jun Cai, Bo Xu, Karen Kie Yan Chan, Xueying Zhang, Bing Zhang, Ziyue Chen, Bing Xu

**Affiliations:** 1 Ministry of Education Key Laboratory for Earth System Modeling, Department of Earth System Science, Tsinghua University, Beijing 100084, China; cai-j12@mails.tsinghua.edu.cn (J.C.); xu-b15@mails.tsinghua.edu.cn (B.X.); cqe15@mails.tsinghua.edu.cn (K.K.Y.C.); 2 Joint Center for Global Change Studies, Beijing 100875, China; 3 Department of Environmental Medicine and Public Health, Icahn School of Medicine at Mount Sinai, New York, NY 10029, USA; xueying.zhang@mssm.edu; 4 School of Public Health (Shenzhen), Sun Yat-sen University, Shenzhen 518107, China; zhangbing4502431@outlook.com; 5 State Key Laboratory of Remote Sensing Science, College of Global Change and Earth System Science, Beijing Normal University, Beijing 100875, China; zychen@bnu.edu.cn

**Keywords:** China, 2009 influenza A(H1N1) pandemic, transport modes, rail travel, spatial spread, quantile regression

## Abstract

There is increasing concern about another influenza pandemic in China. However, the understanding of the roles of transport modes in the 2009 influenza A(H1N1) pandemic spread across mainland China is limited. Herein, we collected 127,797 laboratory-confirmed cases of influenza A(H1N1)pdm09 in mainland China from May 2009 to April 2010. Arrival days and peak days were calculated for all 340 prefectures to characterize the dissemination patterns of the pandemic. We first evaluated the effects of airports and railway stations on arrival days and peak days, and then we applied quantile regressions to quantify the relationships between arrival days and air, rail, and road travel. Our results showed that early arrival of the virus was not associated with an early incidence peak. Airports and railway stations in prefectures significantly advanced arrival days but had no significant impact on peak days. The pandemic spread across mainland China from the southeast to the northwest in two phases that were split at approximately 1 August 2009. Both air and road travel played a significant role in accelerating the spread during phases I and II, but rail travel was only significant during phase II. In conclusion, in addition to air and road travel, rail travel also played a significant role in accelerating influenza A(H1N1)pdm09 spread between prefectures. Establishing a multiscale mobility network that considers the competitive advantage of rail travel for mid to long distances is essential for understanding the influenza pandemic transmission in China.

## 1. Introduction

Four influenza pandemics occurred at intervals of several decades during the past 100 years, the most recent of which occurred in 2009 and was caused by influenza A(H1N1)pdm09 virus [1]. Cases of human infection with influenza A(H1N1)pdm09 virus were first identified in the United States (US) and Mexico in early April 2009 [2]. The rapid global spread of the virus led the World Health Organization (WHO) to raise the influenza pandemic alert level to the highest phase six on 11 June 2009 [3]. On 10 August 2010, the WHO announced that the world had moved into the post-pandemic period [4]. As of 1 August 2010, laboratory-confirmed cases of influenza A(H1N1)pdm09 including over 18,449 deaths had been reported from more than 214 countries or regions worldwide [5]. The actual fatality of the pandemic could be much higher; it was estimated to have caused between 100,000 and 400,000 deaths globally in the first 12 months of the pandemic [6].

Prior to the emergence of influenza A(H1N1)pdm09 virus, a number of studies had assessed the role of air travel in the spread of pandemic and seasonal influenza viruses at global and regional scales [7,8,9,10,11,12]. It is recognized that the global spread of pandemic influenza is largely associated with international air travel, especially during the introduction period. Therefore, following initial detection of influenza A(H1N1)pdm09 virus in North America, numerous studies employed statistical and mathematical models that incorporated air transportation data to explain its global dissemination [13,14,15,16,17,18,19,20]. A multiscale mobility network comprised of long-range airline traffic and short-scale local commutes was also used to approximate the spreading scenarios at a global scale [21,22]. However, controversy remains over whether short-distance commutes or long-range air travel has more influence on regional influenza spread in the US [11,23]. On the one hand, short-distance commutes have been identified as a major driver of between-state influenza spread [12,23,24]; on the other hand, long-range air travel has also been connected to inter-regional spread [10]. Thus, to improve our understanding of the drivers of spread, it is essential to examine the roles of different transport modes in influenza transmission at a regional scale.

Most previous investigations concentrated on the effects of transport modes on influenza spread in the US. However, few studies examined transport modes in a similarly sized country with a much larger population, such as China. In the US, the vast majority of people travel by automobile for shorter distances and by airplane for longer distances. Trains accounted for only 0.74% of passenger-miles traveled in the US in 2009 [25]. In contrast, railway is a common and principal mode for intermediate and long-range travel between cities in mainland China [26]. For example, 5.12% (77.1–86.9%, after removal of the short-distance traffic volume) of the total passengers in mainland China in 2009 were handled by trains [27]. Consequently, models based on human mobility patterns in the US such as the global epidemic and mobility (GLEaM) model [28] may not be suitable for mainland China. Furthermore, the multiscale mobility network used by the GLEaM model does not contain any commuting data from mainland China [21]. Due to the unique transportation system, it is necessary to investigate the roles of different transport modes in the spatial spread of influenza in mainland China. The increased disease surveillance and data availability in the context of the 2009 influenza A(H1N1) pandemic [24] provides a unique opportunity to conduct such an investigation.

After the first confirmed case of influenza A(H1N1)pdm09 that was imported into mainland China on 10 May 2009 [29], the virus spread rapidly. By 5 July 2009, 1040 cumulative confirmed cases—including 758 imported cases and 282 autochthonous cases—were reported in 24 provinces across mainland China [30]. The rapid transmission and substantial impact of the disease on the public health system caused great concern [31]. A few scholars explored the effects of transport modes on spatial transmission at different geographical scales in mainland China. Xiao et al. [32] analyzed the spatiotemporal transmission of influenza A(H1N1)pdm09 via road traffic between counties and towns within Changsha city, and their results showed that inter-county bus stations played an important role in epidemic diffusion. Fang et al. [33] used survival analysis to analyze the impact of travel-related risk factors on the inter-county invasion of influenza A(H1N1)pdm09 in mainland China, and they found that counties close to airports and intersected by highways rather than railways were significantly associated with earlier virus presence. Their findings of an insignificant influence of rail travel on influenza A(H1N1)pdm09 spread may have been due to the county level at which their study was conducted. In contrast, other research suggested that rail travel played an important role in influenza A(H1N1)pdm09 spread across mainland China, including a reported transmission of the virus on a train [34]. Moreover, in the hybrid model developed by Weng and Ni [35] to evaluate the containment and mitigation strategies of influenza A(H1N1)pdm09 in mainland China, trains and airlines were transportation modes for travel between prefecture-level cities, which indicated they were responsible for the large-scale spread of the virus. In response to the conflicting results regarding the effects of different transport modes on influenza transmission, particularly air and rail travel, we examined their roles in the spread of influenza A(H1N1)pdm09 in mainland China at the prefecture level.

In this study, we characterized the spatial variability in arrival and peak times of influenza A(H1N1)pdm09 transmission across the 340 affected prefectures in mainland China based on daily laboratory-confirmed infections from 10 May 2009 to 30 April 2010. We first evaluated the effects of airports and high-ranking railway stations on arrival time and peak time. To quantify the roles of different transport modes in the two spread phases of the pandemic, we fitted quantile regression models to assess the relationships between various quantiles of arrival timing and passenger traffic via air, rail, and road in 115 prefectures with data for all three transport modes.

## 2. Materials and Methods

### 2.1. Epidemiological Data

**Figure 1:**
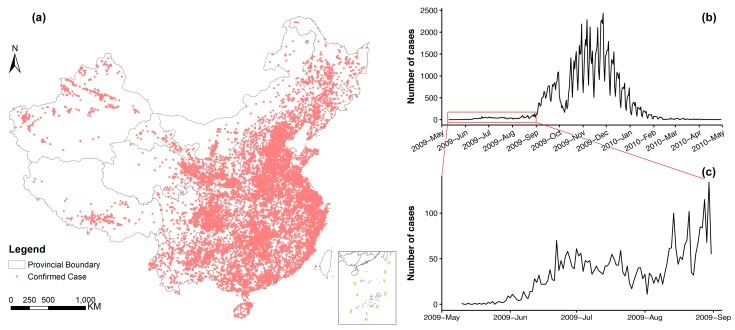
The spatial and temporal distribution of all 127,797 laboratory-confirmed influenza A(H1N1)pdm09 cases reported to the China Information System for Disease Control and Prevention in mainland China from 10 May 2009 to 30 April 2010: (**a**) spatial distribution of geocoded residential addresses; (**b**) the daily epidemic curve from 10 May 2009 to 30 April 2010; (**c**) the enlarged daily epidemic curve from 10 May 2009 to 31 August 2009.

We obtained data from all influenza A(H1N1)pdm09 cases reported to the China Information System for Disease Control and Prevention (CISDCP) from 10 May 2009, when the first confirmed case was reported, to 30 April 2010. These were all classified as suspected and laboratory-confirmed cases. For more details about CISDCP and case definitions, refer to [33]. We only used the laboratory-confirmed cases in our analyses. Case information included but was not limited to case classification, gender, birth date, onset date, diagnosis date, occupation, residential address, work address, and hospital admission address. The residential address of each case was geocoded into latitude and longitude coordinates with the Google Geocoding API [36]. The resulting geographic coordinates were examined at the county level to ensure geocoding quality. The spatiotemporal distribution of the cases is shown in Figure 1.

### 2.2. Passenger Volume Data

Mainland China is comprised of 31 provinces that are further divided into 341 administrative prefectures. The passenger volumes of air, rail, road, and boat travel for each prefecture in 2009 were obtained from the 2010 China City Statistical Yearbook [37]. Because the data were only available for 281 prefecture-level cities, we supplemented passenger volume data for the remaining prefectures by individually looking up the 2009 statistical bulletins on national economic and social development. Finally, of all 340 affected prefectures, 334 (98.2%) had passenger volumes for at least one type of transport mode. Here, only air, rail, and road passenger volumes were included in our analyses, given that boat passenger volumes had the smallest proportion of total passenger volumes (0.7%) and explained the least variance in arrival days defined below (3.2%). The availability of air, rail, and road transport varied across 334 prefectures. All three transport modes were available in 115 (33.8%) prefectures; air and road transport were both available in 20 (5.9%) prefectures, while rail and road transport were both available in 140 (41.2%) prefectures. The automobile was the only transport mode for the remaining 59 (17.3%) prefectures.

### 2.3. Definitions of Arrival Day and Peak Day

**Figure 2:**
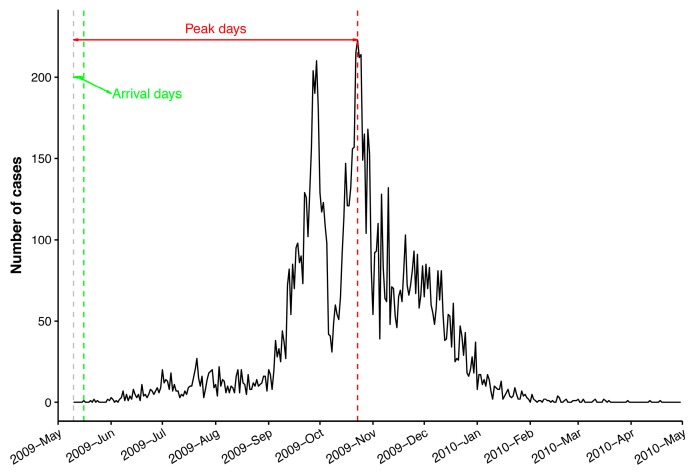
Illustration of arrival days and peak days using the daily epidemic curve of confirmed cases in Beijing. The gray, green, and red vertical dashed lines represent, respectively, the date of the first case in mainland China (10 May 2009), the date of the first case in Beijing (16 May 2009), and the date with the highest incidence in Beijing (23 October 2009).

A daily epidemic curve of newly confirmed cases for each prefecture was generated based on the diagnosis date using the R package incidence [38]. To characterize the inter-prefecture spread of influenza A(H1N1)pdm09 in mainland China, two measures—arrival day and peak day—were derived from the epidemic curve. As defined in [16], arrival day for a given prefecture was defined as the number of days from 10 May 2009 (the date of the first country case) to the date of the first case in each prefecture. Likewise, peak day was defined as the number of days from 10 May 2009 to the date with the highest incidence (Figure 2).

### 2.4. Comparisons of Arrival Days and Peak Days between Prefectures with and without Transport Hubs

**Figure 3:**
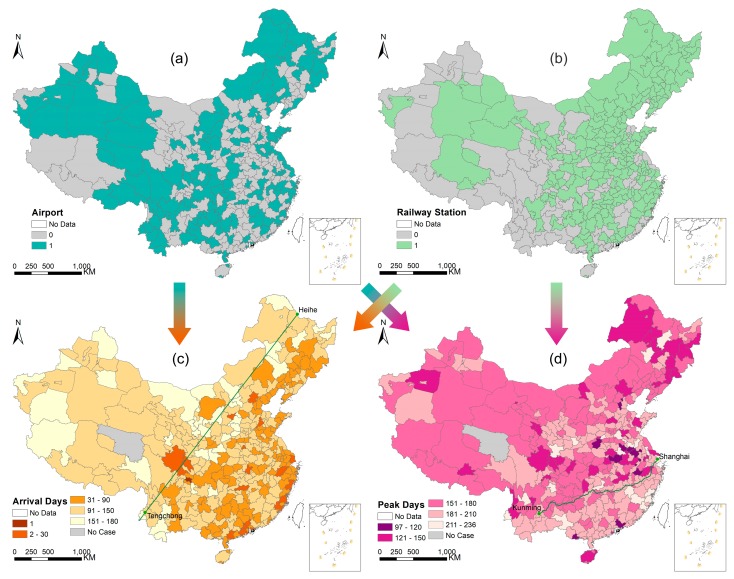
The presence/absence (1/0) of (**a**) airports and (**b**) railway stations in prefectures, and the distributions of (**c**) arrival days and (**d**) peak days for prefectures. The green line in (**c**) indicates the Heihe-Tengchong (Hu) line, and the one in (**d**) indicates the Hukun Railway that connects Shanghai and Kunming. The arrows illustrate that arrival days (or peak days) between prefectures with and without airports (or railway stations) are compared using the Mann-Whitney U test, and their spatial heterogeneity stratified by airports (or railway stations) are detected using *q*-statistic test.

The presence of railway stations is a better proxy of passenger volume than being intersected by railways because there are prefectures that are intersected by railways but do not have railway stations (e.g., Luzhou, a city located in Sichuan province). Hence, we compiled a list of all the airports and railway stations in mainland China at the end of 2009 and identified their locations on the map. The National Railway Administration of China manages railway stations according to their status level, which is determined by the daily arrival, departure, and transfer passenger volume (DPV) as well as by their geographical conditions. Status levels are classified as principal (DPV > 60,000), first-rank (DPV = 15,000–60,000), second-rank (DPV = 5000–15,000), third-rank (DPV = 2000–5000), fourth-rank, or fifth-rank passenger stations. To ensure that prefectures were exposed to intensive railway transportation, only principal, first-, and second-class railway stations were used to indicate exposure in our analyses. All 340 prefectures were assigned to “with airport” or “without airport” categories and “with railway station” or “without railway station” categories based on whether there was an airport or railway station inside the prefecture (Figure 3a,b). To evaluate the respective effects of the presence of airports and railway stations on the inter-prefecture invasion of influenza A(H1N1)pdm09, we used the Mann-Whitney U test at the 95% confidence level to examine whether prefectures with and without a particular transport hub showed a significant difference in arrival day. The same test was also applied to peak day.

The spatial stratified heterogeneity (SSH) of arrival day is realized by the Heihe-Tengchong line (hereafter referred to as the Hu line [39]) (Figure 3c). To measure the degree of the stratified heterogeneity [40], we stratified 340 prefectures based on the relative positions of their administrative centers and the Hu line. Then, the SSH q-statistic for the stratified arrival days was calculated, and the significance of the stratified heterogeneity was also tested with a significance level of 0.05 using the R package geodetector [41]. Using the same procedure, we also assessed the assumption that there is SSH in peak days stratified by the Hukun Railway (Figure 3d). Furthermore, to detect the determinant power of transport hubs to the SSH of arrival day, the q-statistic and corresponding *p* value were also calculated for the airports and railway stations factors, respectively. The same analysis was also applied to peak day.

### 2.5. Quantile Regression of Arrival Days on Passenger Volumes

Because there was large variance in passenger volumes across prefectures, log transformations were conducted on the air, rail, and road passenger volume data to shrink their scales. We performed Pearson’s correlation analysis to identify the associations between log-transformed passenger volumes and arrival days for each transport mode.

**Figure 4:**
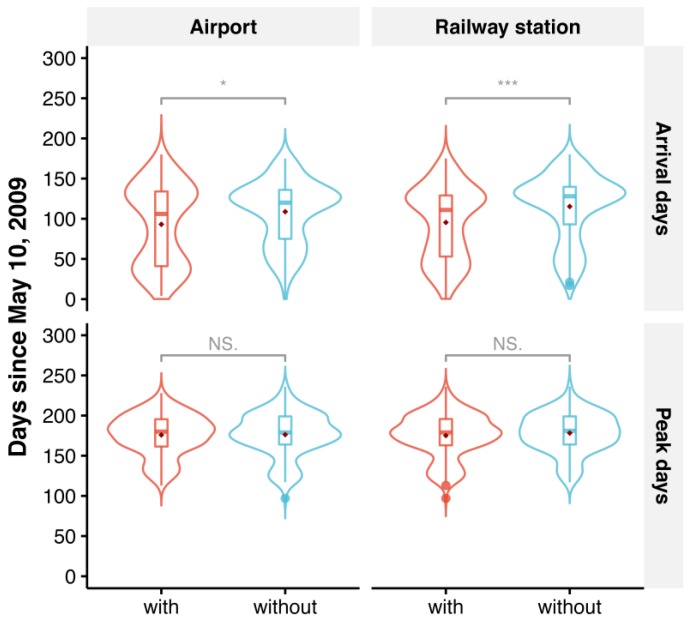
Violin plots of arrival days and peak days between prefectures with (in red) and without (in cyan) airports (or railway stations). Box plots are embedded into violin plots to add summary statistics. The dark red points represent the mean arrival days (or peak days). * *p* < 0.05; *** *p* < 0.001; NS., not significant for comparing arrival days (or peak days) between prefectures with and without transport hubs using a Mann-Whitney U test.

**Figure 5:**
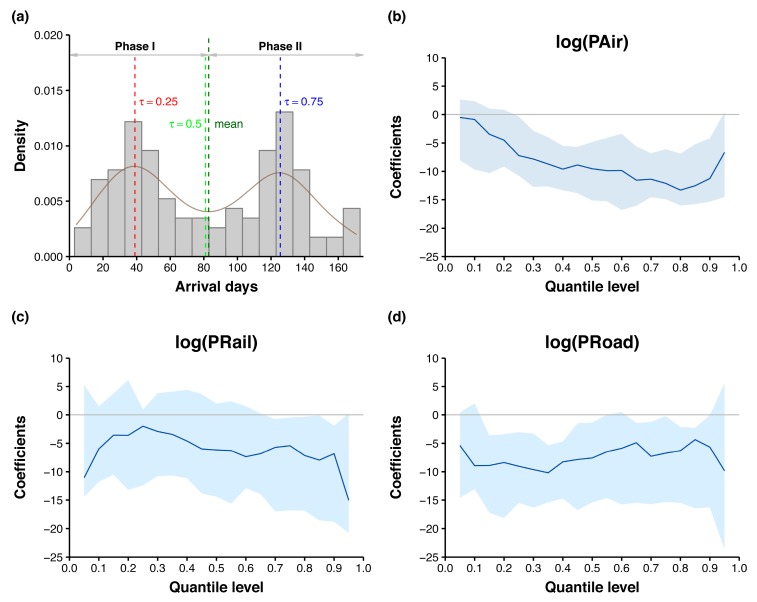
Quantile process regression of arrival days in 115 prefectures. In (**a**) the density plot of arrival days, the red, green, and blue vertical lines indicate, respectively, the τ = 0.25, 0.50, and 0.75 quantiles of arrival days, whereas the dark green vertical line indicates the mean arrival days. In quantile process plots for log-transformed air (**b**), rail (**c**), and road (**d**) passenger volumes, the blue curves and shaded areas represent the quantile regression coefficients and 95% confidence intervals.

To examine the roles of transport modes in shaping the two peaks of the bimodal distribution of arrival days (Figure 4 and Figure 5a), we applied quantile regression to fit specified percentiles of arrival days. Quantile regression, introduced by Koenker and Bassett [42], is distribution agnostic and capable of modeling the entire distribution of the response. In contrast, standard ordinary least squares regression only models the mean of the response. To consider the effect of prefectural locations on the spatial spread of influenza A(H1N1)pdm09, latitudes and longitudes of their administrative centers were also included as covariates. We therefore fitted the following multivariate quantile regression model to assess the associations between different quantiles of arrival days and passenger volumes for multiple transport modes in the 115 prefectures where data on all three transport modes were available:

(1) $$Q_{\tau }\left(y_{i}\right)=\beta_{0}\left(\tau \right)+\beta_{1}\left(\tau \right)Lat_{i}+\beta_{2}\left(\tau \right)Lng_{i}+\beta_{3}\left(\tau \right)\log\left(PAir_{i}\right)\;+\beta_{4}\left(\tau \right)\log\left(PRail_{i}\right)+\beta_{5}\left(\tau \right)\log\left(PRoad_{i}\right),$$

where $y_{i}$ is the arrival day in prefecture i (i=1,…,115), τ is the quantile level, and $Q_{\tau }\left(y_{i}\right)$ is the corresponding conditional quantile of arrival days. $\beta_{0}\left(\tau \right)$ denotes the intercept for quantile level τ. $Lat_{i}$ and $Lng_{i}$ are the latitude and longitude coordinates of the administrative center in prefecture i. $\log\left(PAir_{i}\right)$, $\log\left(PRail_{i}\right)$, and $\log\left(PRoad_{i}\right)$ are the log-transformed air, rail, and road passenger volumes (10,000 persons) in prefecture i, respectively, and $\beta_{1}\left(\tau \right),\ldots ,\beta_{5}\left(\tau \right)$ are the corresponding regression coefficients for quantile level τ.

We separately fitted the quantile regression models for the quantile levels τ = 0.25, 0.50, and 0.75. To fully describe the bimodal distribution of arrival days, quantile process regressions for uniformly spaced values of τ in the interval (0, 1) with an increment of 0.05 were also fitted. The 95% confidence intervals (CIs) for coefficient estimates were calculated using the default “rank” method [43].

The quantile regression was implemented using R package quantreg [44]. All data analyses were performed in R version 3.5.1 [45].

## 3. Results

### 3.1. Summary of Influenza A(H1N1)pdm09 Infections in Mainland China

The first case of influenza A(H1N1)pdm09 in mainland China was confirmed on 10 May 2009 as a Chinese student returning from the United States to Neijiang city, Sichuan province [29]. On 29 May 2009, the first secondary case of influenza A(H1N1)pdm09 in mainland China was confirmed in Guangzhou city, Guangdong province [46]. Also in Guangzhou, the first untraceable autochthonous case of influenza A(H1N1)pdm09 in mainland China was confirmed on 11 June 2009 [47]. As shown in Figure 1c, the number of confirmed cases increased slowly from May 2009 to the end of August 2009 with a small uplift around late June. Starting in September 2009, when the new school term began, the number of confirmed cases increased substantially but decreased sharply during the eight-day National Day holiday (1–8 October 2009). The number of confirmed cases then rebounded at the end of the holiday period and peaked by the end of November 2009 (Figure 1b). By the end of April 2010, a total of 127,797 confirmed cases of influenza A(H1N1)pdm09, including 806 deaths, had been reported to the Chinese Center for Disease Control and Prevention from 340 of all 341 prefectures; only Yushu, a less populated prefecture in Qinghai province, reported no cases (Figure 3c).

### 3.2. Effects of Airports and Railway Stations on Influenza A(H1N1)pdm09 Inter-Prefecture Spread and Peak

The arrival days and peak days for the 340 affected prefectures are summarized in Table 1. The arrival days for the 340 affected prefectures ranged from 1–180 days (median: 119 days, interquartile range (IQR): 61–136 days), while peak days distributed in a narrower range of 97–236 days (median: 179 days, IQR: 163–198 days). Both the maps of arrival days (Figure 3c) and peak days (Figure 3d) showed apparent SSH; most prefectures with earlier arrival days were located in the southeast of the Hu line (q = 0.143, *p* < 0.001), whereas prefectures with relatively late peak times were generally located below the Hukun Railway (q = 0.091, *p* = 0.002). Using a Pearson’s correlation coefficient test, no significant correlation between arrival day and peak day was found (r = −0.08, *p* = 0.13).

The distributions of arrival days and peak days by transport hub presence are shown in Figure 4. The plots of arrival days show a bimodal distribution with two peaks. The first peak of arrival days occurred around 40 days, while the second peak of arrival days occurred around 125 days. The distribution of arrival days was different between prefectures with and without transport hubs. Specifically, the two peaks were of similar intensity for prefectures with a transport hub, whereas the second peak of arrival days was greater than the first for prefectures without a transport hub. The plots of peak days have a less distinctive double-peak feature. The first smaller peak occurred around 125 days, and the second larger peak occurred around 175 days—before the end of November 2009. There was no apparent difference in the distribution of peak days between prefectures with and without airports. However, the distribution of peak days changed less abruptly for prefectures without railway stations than with.

Among the 340 affected prefectures, 155 (45.6%) prefectures with airports were generally affected earlier than the other 185 (54.4%) prefectures without airports (median arrival days: 106 days versus 120 days; Mann-Whitney U test, *p* = 0.005, see Figure 4). In contrast, no statistically significant difference in peak day between these two groups was found (median: 180 days versus 179 days; Mann-Whitney U test, *p* = 0.87, see Figure 4). The railway station results were similar to the airport results. There was a statistically significant difference in arrival day between the 234 (68.8%) prefectures with railway stations and the 106 (31.2%) prefectures without (median: 111 days versus 128 days; Mann-Whitney U test, *p* < 0.001, see Figure 4). By contrast, no statistically significant difference in peak day was detected between these two groups (median: 179 days versus 181 days; Mann-Whitney U test, *p* = 0.42, see Figure 4). The SSH q-statistic showed consistent results with the Mann-Whitney U test. There was significant stratified heterogeneity in arrival day for both stratifications of airports (q = 0.031, *p* = 0.001) and railway stations (q = 0.042, *p* = 0.002), whereas no significant stratified heterogeneity in peak day was detected for the stratifications of airports (q = 0.000, *p* = 0.897) or railway stations (q = 0.003, *p* = 0.817). The difference in median arrival day between prefectures with and without transport hubs was ~2 weeks (120 days versus 106 days for airport; 128 days versus 111 days for railway station). Additionally, there was approximately a one week lag in median arrival day when comparing the corresponding analysis of railway stations with airports (111 days versus 106 days for prefectures with transport hubs; 128 days versus 120 days for prefectures without transport hubs). By comparison, the median peak was around 180 days irrespective of the presence of transport hubs in the prefectures (Table 1).

### 3.3. Roles of Transport Modes in Inter-Prefecture Spread of Influenza A(H1N1)pdm09

The correlations between arrival days and log-transformed passenger volumes by transport modes are presented in Table 2. For each transport mode, log-transformed passenger volumes were negatively correlated with arrival days across prefecture groups, but the relationships were significant in all prefecture groups except for the 20 prefectures with only air and road transport (r = −0.32, *p* = 0.17 for air travel; r = −0.34, *p* = 0.14 for road travel).

As previously shown in Figure 4, mainland China experienced two distinct phases of inter-prefecture spread of influenza A(H1N1)pdm09. To examine the roles of different transport modes in these two spread phases, we focused our analyses on the 115 prefectures where all three transport modes were available. As shown in Figure 5a, the distribution of arrival days in 115 prefectures was bimodal with two peaks at approximately 40 (20 June 2009) and 125 (15 September 2009) days. The respective peaks coincided with a small uplift (Figure 1c) and a sharp increase (Figure 1b) in the daily influenza A(H1N1)pdm09 incidence in mainland China. These two phases (I and II) with peaks at the 0.25 and 0.75 quantiles of arrival days were split by the 0.50 quantile (80 days since 10 May 2009, i.e., approximately 1 August 2009) (Figure 5a). The associations between the 0.25, 0.50, and 0.75 quantiles of arrival days and passenger volumes for the different transport modes in 115 prefectures are presented in Table 3. Irrespective of the quantile level τ, latitude coordinates for affected prefectures were significantly positively associated with arrival day, whereas longitude coordinates were significantly negatively related to arrival day. After adjusting for the geographic locations of prefectures, both air and road passenger volumes in log-scale were significantly negatively associated with arrival day for τ=0.25, 0.50, and 0.75. By contrast, log-transformed rail passenger volumes were also negatively associated with arrival day across the three quantile levels; however, the association was significant only for τ=0.75 (regression coefficient = −5.42, 95% CI: −16.80, −0.45). As indicated by the pseudo R^2^, the quantile regression model for τ=0.50 explained the most variance in arrival days (45%).

The quantile process plots of the three transport modes shown in Figure 5 further demonstrate the change in quantile regression coefficients and 95% CIs as a function of quantile level τ. As τ increases, the regression coefficient of log-transformed air passenger volume decreases, whereas the regression coefficient of log-transformed rail passenger volume increases slightly before τ = 0.25, then decreases. Yet, the regression coefficient of log-transformed road passenger volume is constant prior to τ = 0.4, then increases slightly with the increase of τ. We further observed that the negative associations between log-transformed air passenger volume and the lower quantiles of arrival day appear to be insignificant because the upper confidence limits are greater than 0 for quantile levels less than 0.25. Likewise, log-transformed rail passenger volumes significantly negatively affected ≥0.70 quantiles of arrival days.

## 4. Discussion

To understand the roles of different transport modes in the spread of the 2009 influenza A(H1N1) pandemic between prefectures across mainland China, this work used arrival day and peak day to characterize the pandemic spread and evaluated the influence of travel-related factors on it. During the entire invasion period of the virus, road travel consistently played a significant role. Rail travel played an insignificant role during phase I but significantly affected the inter-prefecture spread during phase II. The role of air travel became more important as the virus spread.

### 4.1. Two Phases and Direction of Influenza A(H1N1)pdm09 Spread between Prefectures

While a pronounced double-wave feature in the daily epidemic curve was barely noticeable prior to the National Day holiday, the remarkably bimodal distribution of arrival days suggests that the inter-prefecture spread of influenza A(H1N1)pdm09 across mainland China had two distinct phases that were split at approximately 1 August 2009. Interestingly, the phase I of spatial spread coincided with the early containment phase of the 2009 pandemic when an individual case-based surveillance was implemented until mid-July 2009 [48]. Our results suggest that containment measures successfully suppressed the increase in influenza A(H1N1)pdm09 incidence during phase I but failed to restrict its spatial expansion. Consequently, individual case-based surveillance was terminated by mid-August 2009 [48], which made the inter-prefecture of influenza A(H1N1)pdm09 easier during phase II. Additionally, according to the associations between school openings and influenza A(H1N1)pdm09 transmission found in the US [49,50], school openings in early September 2009 may have also contributed to the second peak of influenza A(H1N1)pdm09 spread between prefectures.

Quantile regression analyses showed that the arrival time of influenza A(H1N1)pdm09 in an individual prefecture was always significantly negatively and positively associated with prefectural longitudes and latitudes, respectively, regardless of spread phase. These results suggest that the virus generally spread from the southeast to the northwest of mainland China, which confirms previous findings [33]. The observed direction of spatial spread reflected the fact that, during the early containment phase, a large proportion of international-travel related cases were imported into mainland China via international airports in eastern coastal cities, particularly those in Guangdong and Fujian provinces [33,48]. From there, the infection was disseminated to the other parts of mainland China. This fact was also partially responsible for the earlier presence of influenza A(H1N1)pdm09 in prefectures located in the southeast of the Hu line. Another factor strongly related to this phenomenon was the obvious spatial heterogeneity of the population distribution stratified by the Hu line. In the southeast of the Hu line, 93.9% of the population in 2015 live in 42.8% of the area, and the population density is 314.9 people/km^2^, 20.5 times that of the other side [51]. As a result, significant stratified heterogeneity of arrival day was detected for the stratification of the Hu line, whose determinant power was as much as 14.9%.

### 4.2. Impact of Transport Hubs on Arrival Day and Peak Day

The lack of significant correlation between arrival day and peak day among the 340 affected prefectures indicates that the early arrival of influenza A(H1N1)pdm09 was not associated with early peak incidence as one might expect. Specifically, the peaks in prefectures above the Hukun Railway were concentrated before the end of November 2009, which was approximately two months earlier than the typical peak of seasonal influenza epidemics in Northern China (January–February) [52]. This is because, as a novel virus to which humans have little immunity, influenza A(H1N1)pdm09 is associated with high mortality and can spread more quickly than mild seasonal influenza epidemics [12]. It is interesting to note that the apparent difference in peak time of the influenza pandemic was delineated by the Hukun Railway rather than 27° N, which was suggested by Yu et al. [52] for identifying epidemiological regions characterized by distinct influenza seasonality in China. This discrepancy may be because Yu et al. conducted their analysis at the province level by aggregating sentinel hospital-based influenza surveillance data. This emphasizes the need to characterize the influenza seasonal patterns in China at the prefecture level.

The presence of airports or high-ranking railway stations in prefectures significantly advanced arrival day but had no evident impact on peak day. This finding is also confirmed by the SSH *q*-statistic test; 3.1% and 4.2% of the SSH of arrival day were attributed to airports and railway stations. On the contrary, the almost zero determinant powers of both airports (q = 0.000) and railway stations (q = 0.003) to peak day suggest that the spatial heterogeneity of peak day was not associated with the presence of transport hubs. A possible explanation for this difference might be that, because airports and railway stations are suitable proxy variables for air and rail travel, the arrival of passengers increased the probability of transmitting influenza A(H1N1)pdm09. However, once these prefectures were affected, peak of transmission within a prefecture was more likely to be determined by local environmental factors such as humidity and temperature [52,53,54]. More specifically, experimental studies indicate that aerosol transmission of influenza A(H1N1)pdm09 is sensitive to temperature and humidity [55]. Our previous analyses also suggested that absolute humidity was the dominant meteorological factor associated with spatial spread of influenza A(H1N1)pdm09 across mainland China [56]. Furthermore, prefectures with airports or high-ranking railway stations generally have high population density and greater mobility. Therefore, the approximate two week lag in arrival day between prefectures with and without transport hubs suggests that influenza A(H1N1)pdm09 might spread rapidly and hierarchically among populous cities and then to less populated areas across mainland China, which may resemble the spatial spread patterns previously described for seasonal flu in the US [12]. This proposed pattern is supported by a study on human travel patterns in mainland China, which also suggested that a pandemic emerging in more developed areas might be expected to spread more rapidly [57]. In addition to timing, a recent study indicated that urbanization and humidity have strong influences over the epidemic intensity of influenza [58].

### 4.3. Roles of Transport Modes in Inter-Prefecture Spread of Influenza A(H1N1)pdm09

The negative quantile regression coefficients of air and rail passenger volume on arrival day supported our hypothesis that air and rail travel accelerated the inter-prefecture spread of influenza A(H1N1)pdm09 across mainland China. The most interesting finding was that both air and road travel played a significant role in accelerating influenza A(H1N1)pdm09 spread between prefectures across phases I and II, whereas the coefficient for rail travel was only significant during phase II. This finding could be explained by the fact that after entering mainland China, numerous international travel-related cases continued to travel back to their hometowns by air and road. Thus, the role of rail travel was not apparent during phase I. Meanwhile, students returned to school at the start of the new term in early September 2009, and increased student mobility led to the increase in influenza A(H1N1)pdm09 transmission at that time. In particular, college students mostly undertook trans-city travel, for which railway is the dominant transport mode in mainland China [26]. Thus, the role of rail travel became more important during phase II. To our knowledge, the dynamic effects of transport modes on influenza A(H1N1)pdm09 spread only were reported for road traffic in a local study conducted in Changsha city [32].

Regarding the roles of air and road travel in the spatial spread of influenza A(H1N1)pdm09 across mainland China, our results are consistent with [33]. However, it should also be noted that our findings on the role of rail travel is contrary to that of Fang et al. [33], who found an insignificant association between counties intersected by railways and virus arrival. Despite the different transport-related variables and statistical methods used, this inconsistency may be mainly attributed to the difference in spatial scales of investigation. Our study was conducted at the prefecture level, whereas theirs were carried out at the county level. As noted by Dai and Jin [26], transport modes have different competing advantages for different distances, and railway is the dominant mode for intermediate and long-range travel between cities in mainland China. Therefore, the distance between counties is too short to examine the role of rail travel in the spatial spread of influenza A(H1N1)pdm09. Instead, assessing roles of different transport modes at the prefecture level is appropriate because it can avoid two situations—the coarse data available at the provincial level would inadequately describe the spatial spread of the pandemic, and the insufficient number of cases at the county level would result in unreliable epidemic curves that are used to derive the spatial patterns of pandemic spread. Furthermore, the difference in spatial scales of investigation may also partially explain the controversy between Viboud et al. [12] and Brownstein et al. [10] regarding the drivers of seasonal influenza spread in the US. It can therefore be suggested that influenza propagation is driven by different transport modes at different spatial scales; while air travel plays a role in long-range dissemination across regions, between-state or even between-city spread is driven by short-distance commutes.

### 4.4. Limitations and Prospect

Our study had several limitations. First, due to resource limitations in case identification and outbreak investigation, the reporting criteria for case-based surveillance changed from individual cases regardless of clinical severity to hospitalized cases by mid-August 2009 [48]. Arrival days after mid-August 2009 tended to be biased because mild and asymptomatic patients might not have sought hospital care. The underreported cases during the later stage of the pandemic, particularly the large drop in case number due to the National Day holiday, may also have influenced peak day estimation. Moreover, annual passenger traffic data available for 2009 were used to examine the roles of transport modes in the inter-prefecture invasion of influenza A(H1N1)pdm09 for May–November 2009. Using monthly transportation data from the invasion period, or even data corresponding to the quantiles of interest for arrival day, could improve model performance. Finally, although regression coefficients for the different transport modes can be directly compared based on their magnitudes, we were unable to draw a definite conclusion about the relative importance of different transport modes for accelerating the inter-prefecture spread of influenza A(H1N1)pdm09.

To address such a challenge, a multiscale mobility network based on human mobility patterns in China needs to be established. The mobility network should be comprised not only of long-range airline and short-distance road traffic flows but also of intermediate and long-range rail travel flows. Additionally, sophisticated mathematical models with considerations given to administrative hierarchy of population and human travel rules should also be developed to better simulate the spread of pandemic influenza across China. The hybrid model combining meta-population and agent-based models proposed by Weng and Ni [35] seems to be a direction worth exploring further in future research.

## 5. Conclusions

We conclude that, in addition to air and road travel, rail travel also played a significant role in accelerating the inter-prefecture spread of influenza A(H1N1)pdm09 across mainland China. Our study provides evidence that the role of different transport modes in the spatial spread of influenza should be evaluated at the appropriate spatial scale. Our findings suggest that establishing a multiscale mobility network that considers the unique competitive advantage of rail travel for mid to long distances is essential to understanding pandemic influenza spread and to informing control strategies for future pandemics in China.

## Figures and Tables

**Table 1:** Summary statistics of arrival days and peak days for 340 affected prefectures in mainland China.

Category		Arrival Day ^a^					Peak Day				
		Min	Q1	Median	Q3	Max	Min	Q1	Median	Q3	Max
**All** (340)		1	61	119	136	180	97	163	179	198	236
**Airport**	With (155)	4	41	106	134	180	113	162	180	196	228
	Without (185)	1	75	120	136	175	97	164	179	199	236
**Railway station**	With (234)	1	53	111	129	175	97	163	179	196	236
	Without (106)	17	93	128	140	180	117	164	181	199	236

^a^ Arrival day and peak day are calculated as starting from 10 May 2009, the date of the first confirmed case reported in mainland China.

**Table 2:** Pearson correlation coefficients between arrival days and log-transformed passenger volumes by transport modes in 334 prefectures.

Transport Modes (No. of Prefectures)	log(*PAir*)	log(*PRail*)	log(*PRoad*)
Air + Rail + Road (115)	−0.58 ***	−0.47 ***	−0.60 ***
Air + Road (20)	−0.32	-	−0.34
Rail + Road (140)	-	−0.17 *	−0.25 **
Road (59)	-	-	−0.54 ***

Note: log(*PAir*), log(*PRail*), and log(*PRoad*) are the log-transformed air, rail, and road passenger volumes (10,000 persons) in each prefecture. * Correlation coefficient is significant at the 0.05 level (2-tailed), ** for 0.01, and *** for 0.001. All values are rounded to two decimal places.

**Table 3:** Multivariate quantile regression showing the associations between the 0.25, 0.50, and 0.75 quantiles of arrival days and passenger volumes of three transport modes in 115 prefectures.

Variables ^a^	τ = 0.25	τ = 0.50	τ = 0.75
Intercept	274.08 (222.13, 344.09) ^b^	295.84 (201.19, 381.45)	256.25 (189.21, 457.03)
*Lat*	1.95 (1.30, 3.69)	1.78 (1.36, 3.34)	2.29 (1.00, 4.06)
*Lng*	−1.38 (−1.83, −0.46)	−1.15 (−2.22, −0.55)	−0.80 (−2.33, −0.33)
log(*PAir*)	−7.23 (−10.75, −0.38)	−9.54 (−15.13, −4.81)	−12.10 (−14.90, −6.12)
log(*PRail*)	−1.99 (−12.41, 0.97)	−6.18 (−14.41, 1.99)	−5.42 (−16.80, −0.45)
log(*PRoad*)	−9.04 (−15.52, −3.05)	−7.58 (−15.41, −1.37)	−6.73 (−15.35, −0.23)
R^2 c^	0.33	0.45	0.41

^a^*Lat* and *Lng* are prefectural latitude and longitude coordinates. log(*PAir*), log(*PRail*), and log(*PRoad*) are the log-transformed air, rail, and road passenger volumes (10,000 persons) in each prefecture. ^b^ All regression coefficients are rounded to two decimal places. Numbers in parentheses are 95% confidence intervals. ^c^ Pseudo R^2^ are reported for quantile regression.

## Data Accessibility

The data and analysis code for this study have been made publicly available under MIT license at the GitHub repository, https://github.com/caijun/H1N1Transport.
